# Author Index for Volume 93

**DOI:** 10.1038/sj.bjc.6602897

**Published:** 2005-12-06

Aaboe, M 1182

Aamdal, S 749

Abbruzzese, JL 195

Abdi, E 1236

Ackland, SP 1236

Adami, H-O 493

Adamo, V 896

Adamson, P 811

Aebi, S 709

Agelidou, A 763

Agelidou, M 763

Aggarwal, BB 70

Agnantis, NJ 1382

Aherne, W 868, 876

Ahlbom, A 842

Ahlgren, J 515

Ahlman, H 1144

Aikou, T 688

Aitini, E 7

Akechi, T 1329

Akslen, LA 933

Albanell, J 1285

Al-Baradie, RS 1277

Alcover, J 1285

Alexanian, R 70

Al-Hindi, H 1277

Ali, IU 953

Allen, NE 582

Altman, DG 387

Alvarez, E 1305

Amici, A 1250

Amini, R-M 515

Amit-Vazina, M 70

Andersen, A 159

Andrieu, N 730

Androulakis, N 763

Angeli, A 633

Antinori, A 1084

Antoniou, D 1106

Anttila, A 862

Anumanthan, G 1157

Aoki, D 999

Aoki, K 441

Apolone, G 504

Appleman, LJ 54

Arakawa, T 331

Araki, J 770

Arcangeli, A 781

Arigami, T 688

Arima, H 688

Armenaki, O 1106

Armstrong, S 41

Arquette, MA 46

Arribas, J 1267

Arthur, A 41

Ashford, M 1202

Astoul, P 450

Athanasiadis, A 763

Auvinen, A 842

Avcu, F 267

Axelsson, CK 167

Baas, PC 1122

Bäcklund, LM 124

Baek, JH 1117

Baldi, A 1084

Baldwin, DR 905

Ball, DL 652

Ball, P 1202

Bälter, KA 493

Bang, B 538

Barbieri, F 29

Bardadin, K 144

Barlési, F 450

Barletta, E 185

Baron, JA 838

Barrett-Lee, P 1215

Bartlett, JB 613

Bartolini, S 29, 1334

Basher, W 552

Basolo, F 453

Bastholt, L 273

Bate, S 868

Batistatou, A 1382

Beamer, WG 1137

Becchetti, A 781

Bégin, LR 1019

Begum, M 116

Beijnen, JH 1222

Belcheva, A 233

Bell, KM 98

Bemis, L 1334

Benepal, T 868

Bengala, C 35

Beral, V 817

Bergkvist, L 1310

Berglund, G 582

Bergsland, EK 195

Berlingieri, MT 464

Bernardini, N 319

Bernaudin, JF 1175

Bernhardt, P 1144

Bernstein, L 364

Berrington de González, A 590

Berrino, F 582

Berruti, A 633

Berta, GN 1250

Bertele', V 504

Bertrand, D 1098

Bessaguet, C 107

Betticher, DC 719

Bhagavathula, N 1364

Bianco, AR 896

Bighin, C 7

Bigras, G 575

Bilim, V 544

Billingham, L 1324

Bingham, S 582

Birch-Machin, MA 271

Bjørge, T 807

Blaasaas, KG 842

Blackwell, KL 1350

Block, G 379

Blomqvist, C 515

Blum, KA 46

Bocci, G 319

Boeing, H 582

Boffetta, P 159

Boggi, U 35

Bohlken, A 310

Boice Jr, JD 1038

Bojesen, SE 167

Boldrini, L 453

Bonardi, S 406

Boneberg, E-M 793

Booth, L 622

Booton, R 719

Børresen-Dale, A-L 260, 732

Borysiewicz, LK 1068

Borzomati, D 1084

Bosma, AJ 924

Bottini, A 406

Bouchier-Hayes, DJ 224

Boulos, PB 472

Bowers, NL 719

Bowtell, DDL 310

Boxall, J 979

Bozonnat, M-C 1098

Bramhall, SR 1024

Brammer, RD 1024

Braybrooke, JP 60

Bredenkamp, R 190

Bregård, A 260, 732

Brennan, P 159

Brenner, DE 735

Brenner, H 372

Brewster, DH 159

Brittinger, G 939

Broekhuizen, R 1388

Brohet, R 287

Brøndum-Nielsen, K 260, 732

Bruce, WR 639

Bruland, ØS 81

Brunton, L 1011

Bruzzi, P 7, 733

Bryant, M 1202

Bryant, PE 1038

Buccoliero, AM 781

Buffler, PA 379

Bunn Jr, PA 1334

Burkman, RT 364

Caleo, A 464

Callaghan, R 302

Camacci, T 453

Cameron, D 1112

Camidge, DR 208

Campo, E 1285

Campos, SM 54

Cancellieri, A 1334

Cappuzzo, F 29, 1334

Caraglia, M 611

Carbone, DP 1157

Cardis, E 842

Caricato, M 1084

Carlini, P 185

Carlomagno, C 896

Casabonne, D 1305

Castagneto, B 7

Castaldini, L 29

Castañón, C 1230

Castro, J 1230

Catt, S 1092

Cavaciuti, E 730

Cavallo, F 1250

Cavazzini, G 7

Cei, M 266

Cemazar, M 98

Ceresoli, GL 1334

Ceribelli, A 29

Chae, YS 1117

Chakrabarty, S 1364

Chakraborty, R 1038

Chang, B 493

Charalabopoulos, KA 1382

Chastagner, P 529

Chen, B 1307

Chen, C-H 602

Chen, W 392

Chen, W-F 458

Chen, YC 1057

Cheongsiatmoy, JA 1244

Chetaille, B 450

Cheung, B 310

Chew, GK 1301

Chiang, CI 1057

Chilvers, C 817

Chong, G 510

Choy, STB 1077

Christensen, IJ 273

Chuah, K-L 23

Chung, F-M 602

Ciampor, F 1267

Cigolari, S 611

Cirocco, M 639

Clamp, A 647

Clamp, AR 967

Clark, GM 387

Clark, J 493

Clark, JW 54

Clarke, NW 670

Clemons, M 1152

Cloos, J 1388

Coates, AS 1319

Codony-Servat, J 1285

Cold, S 627

Colin, D 159

Colinet, B 1098

Collatz Christensen, H 842

Collecchi, P 319, 406

Collet, JF 1175

Collette, L 1209

Collins, VP 124

Colpaert, CG 1128

Colucci, G 611

Conan-Charlet, V 107

Concin, H 1062

Condron, CM 224

Conroy, J 699

Conte, PF 35, 406

Contu, A 7

Cook, PA 178

Cooper, CS 478

Copeland, D 1202

Coppola, R 1084

Corbishley, CM 478

Cortesi, E 896

Couanet, D 529

Coursaget, P 1305

Courtenay-Luck, N 1257

Couto, E 1307

Cox, A 1092

Cox, K 41

Cozacov, C 552

Cranston, D 346

Cranston, DW 890

Creutzig, U 1388

Crino, L 29, 406, 1334

Crossley, B 817

Crucitta, E 29

Cruickshank, ME 1301

Crul, M 1222

Cullen, MH 178

Cunningham, D 510, 740

Cunningham, MP 1316

Cuq, P 216

Curwen, GB 1038

Czarnocka, B 144

Dabrosin, C 1005

Daehler, MA 1202

Dai, H 924

Daling, JR 364, 1046

d’Almeida, AK 730

Damato, BE 1191

Damdinsuren, B 557

D’Amico, M 7

Danesi, R 35, 319

Dangoor, A 670

D’Arcy, JA 979

Dashkovsky, I 552

Datta, PK 1157

Daurès, J-P 1098

Davies, FE 269

Davies, SL 60

de Bock, GH 1122

De Giorgi, U 412

de Lange Davies, C 81

De Marinis, F 29

de Marval, F 575

de Mulder, PHM 1209

de Munck, L 1122

De Paoli, P 834

De Placido, S 896

de Prijck, L 1209

de Roos, MAJ 1122

De Vita, F 896

De Vivo, I 804

de Vries, EGE 520

de Vries, J 520, 1122

de Wit, R 1209

Dean, M 953

Deapen, D 364

Dearnaley, D 478

Dearnaley, DP 200

Decaussin-Petrucci, M 464

Del Chiaro, M 35

Del Mastro, L 7

Del Prete, S 611

Del Tacca, M 35, 319

DeLaney, TF 849

Delgado, B 338

Delgado, J 338

Delioukina, M 1202

Dell’Omodarme, M 453

Demirer, T 412

Denes, A 46

Deonarain, MP 1257

Depeille, P 216

Depisch, D 744

Desai, B 1202

Devonec, M 242

Dewey, F 1244

Dewhirst, MW 1350

Dhar, S 483

Dhillon, H 1236

Di Carlo, F 1250

Di Costanzo, F 185, 733

Di Lorenzo, N 781

Dickson, JLB 510

Diem, G 1062

Dietzfelbinger, H 190

Dillner, J 834

Dimitriadis, E 152

Dimitroulis, J 1106

Dimitrov, S 662

Dini, G 412

Dirix, LY 1128

Djafari, L 529

Djamgoz, MBA 1197

Doddoli, C 450

Dogliotti, L 633, 733

Dolsma, WV 520

Domenichini, I 1334

Domingo-Domenech, J 1285

Donati, S 406

Donati, V 453

Dondon, M-G 730

Doni, L 185

Dono, K 557

Donskov, F 757

Dorward, AM 1137

Doz, F 529

Droz, J-P 909

Ducore, JM 379

Dueland, S 749

Duffy, TM 1137

Dunlop, D 1112

Dunlop, DJ 977

Duric, VM 1319

Düring, M 627

Duyster, J 190

Dworan, N 744

Dyrskjøt, L 1182

Eatock, M 1112

Echeta, C 60

Eder Jr, JP 54

Eeles, R 478

Eggo, MC 1024

Eguchi, F 999

Eguchi, H 557

Ehi, K 688

Eikenes, L 81

Eisen, T 825

Ekpo, G 1068

El-Helw, LM 620

Elkan, R 41

Ellis, LM 233

Endoh, M 1395

Engeland, A 807

Engels, FK 173

Epenetos, AA 1257

Escudero, P 1230

Esposti, RD 406

Estrov, Z 70

Evans, DB 233

Evans, LS 620

Evrard, A 216

Ewertz, M 627

Fajac, A 1175

Falcone, A 733

Falconer, A 478

Falk, S 993

Falkner, KL 699

Fallowfield, L 1092

Fallowfield, LJ 1319

Fan, F 233

Fancher, KS 1137

Fanelli, F 611

Fanshawe, T 1295

Farid, NR 1277

Farris, A 896

Faviana, P 453

Fay, K 1364

Faÿnel, J 242

Fears, CL 46

Febbraro, A 611

Feliu, J 1230

Ferguson, DJP 302

Fernández, PL 1285

Ferrari, E 611

Ferrer, B 1285

Fey, MF 709

Feychting, M 842

Fiander, AN 1068

Fich, A 338

Ficorella, C 896

Fioravanti, A 319

Fischer, D 137

Fisher, C 478

Fizazi, K 909

Flanagan, E 60

Fléchon, A 909

Fleming, D 699

Fode, K 757

Folger, SG 364

Fong, K-W 279

Fonrose, X 1356

Fontana, A 35

Fontana, E 35

Fontanini, G 453

Forman, D 597

Formato, R 611

Forni, G 1250

Forrest, LM 977

Forssell-Aronsson, E 1144

Fosså, SD 1209

Foster, JR 208

Fracasso, PM 46

Franceschetti, S 781

Franciosi, V 29

Franklin, WA 1334

Frappaz, D 529

Frassineti, G 733

Freeth, MG 248

Frenkel, EP 1029

Friend, PJ 890

Fryknäs, M 483

Fujii, M 884

Fujisawa, T 1029

Fujita, Y 607

Fujiwara, K 999

Fujiwara, M 799

Fuller Jr, AF 54

Fürstenberger, G 793

Fusco, A 464

Gabriel, B 694

Gallick, GE 233

Gallimore, PH 248

Gallina, P 781

Gallo, C 896

Ganesan, TS 60

Garattini, S 504

Garmy-Susini, B 855

Garvin, S 1005

Gascon, P 1285

Gasperoni, S 185

Gatter, K 1318

Gatter, KC 1168

Gaudernack, G 749

Gaunt, C 1324

Gautschi, O 719

Gazdar, AF 1029

Gebbia, N 896

Geertsen, P 273

Gehl, J 273

Geiger, AM 392

Gelder, CM 1068

Gemini, M 896

Gennari, A 406

Gentet, J-C 529

Geoerger, B 529

Geoffray, A 529

Georgoulias, V 763

Geradts, J 699

Gerson, A 338

Gescher, AJ 735

Gheber, L 338

Ghersi, D 293

Ghiringhello, B 376

Giacca, A 639

Gibault, L 107

Gibson, BES 1388

Giddings, I 1191

Gietema, JA 1356

Gilham, DE 670, 1085

Gion, M 387

Giordano, L 376

Giotta, G 611

Giovannetti, E 35

Giovannucci, E 804

Gislefoss, RE 834

Gitsch, G 694

Glas, AM 924

Gleave, ME 1019

Gleeson, FV 890

Glynne-Jones, R 993

Goh, C 279

Goldberg, RM 1236

Golding, BT 1011

González, E 1230

González-Barón, M 1230

Goodman, A 54

Goodner, SA 46

Gore, M 868

Gorzegno, G 633

Goto, C 15

Gray, MJ 233

Greenwald, P 953

Griffiths, RW 670

Grigoratou, T 1106

Grönberg, H 493

Groth, J 699

Growcott, JW 208

Gruenberger, T 744

Grutsch, JF 1202

Guan, X-Y 137

Guarneri, V 35, 406

Guldberg, P 1038

Gullbo, J 483

Gullo, G 406

Gupta, D 1202

Gustafsson, MG 483

Haas, NB 1356

Haber, M 310

Hackshaw, A 1215

Hadary, A 552

Hagiwara, A 131

Hague, J 961

Hahnemann, JMD 260, 732

Hakama, M 862

Håkansson, N 1310

Hakulinen, T 372

Halder, SK 1157

Hall, PA 499

Hall-Craggs, MA 961

Hallmans, G 834

Hamazaki, K 1329

Hamilton, W 399

Hammarström, L 260, 732

Hammerstad, H 749

Hancock, BW 418, 620

Hannah, A 876

Hara, N 544

Hardcastle, A 868, 876

Hardee, ME 1350

Harousseau, JL 412

Harper, P 178

Harris, AL 346, 385, 1128, 1168

Harris, D 70

Hart, KW 1068

Hasan, J 647

Hasegawa, Y 884

Hasenburg, A 694

Hauser, M 749

Hawkins, RE 670, 1085

Hayashi, T 678

Hayes, A 1011

Hayes, C 876

Haynes, R 620

He, YD 924

Hedelin, M 493

Heighway, J 719

Hemminki, K 159, 1307

Hennig, M 190

Hentrich, M 190

Hepworth, SJ 842

Hermon, C 817

Hesse, E 565

Hibi, K 799

Hickson, ID 60

Hill, A 740

Hill, ME 510

Hill, SA 98

Hine, AV 774

Hirohashi, S 1382

Hirose, K 15

Hirsch, FR 1334

Hiscott, PS 1191

Hoban, PR 719

Hoffmann, K 582

Hofmann, G 781

Højgaard, L 538

Hojo, M 1341

Hokita, S 688

Holloway, AJ 310

Homma, N 1395

Honda, T 678, 1395

Hopfer, O 709

Horsman, JM 418

Horwich, A 200, 1209

Hou, W 46

Houghton, J 1319

Houlston, RS 825, 1191

Howell, A 1152

Howes, A 1222

Hrushesky, WJM 1202

Hsieh, F-C 1372

Huang, H 699

Hubeek, I 1388

Huddart, R 178

Huddart, RA 200

Huff-Adams, S 1202

Hughes, AM 208

Hughes, S 1191

Humphreys, J 1191

Hurbain, I 1175

Hwang, J 195

Iacopetta, BJ 946

Iaffaioli, RV 611

Iannace, C 896

Ianniello, G 611

Ianniello, GP 896

Ichikawa, D 131

Ichikawa, S 607

Ichimura, K 124

Ichinose, Y 770

Iizasa, T 1029

Ikarashi, Y 441

Illing, RO 890

Imaeda, N 15

Inagaki, M 1329

Incoronato, P 611

Innes, H1324

Inoue, M 331

Inoue, Y 770

Iop, A 185

Isaacs, W 493

Isaksson, A 483

Ishigami, S 688

Ito, H 15, 131

Ito, K 799

Ito, S 986

Itoh, K 770

Itoi, T 544

Ivarsson, K 435

Ivy, SP 46

Iwai, N 607

Iwamoto, N 770

Iwata, H 15

Izumi, S 1341

Jackman, A 868

Jackson, DG 1168

Jacot, W 1098

Jäger, M 694

Janin, N 730

Jansner, K 435

Janssen, D 939

Jansson, C 859

Jayson, GC 647, 967

Jenkins, V 1092

Jensen, KM-E 1182

Jeon, SB 1117

Jhavar, S 478

Jobo, T 999

Joel, S 60

Johansen, C 842

Johanson, V 1144

Johansson, J-E 493

Johnsen, SP 838

Johnstone, D 208

Jonasson, JG 159

Jones, M 1236

Joppi, R 504

Juárez, F 1230

Judson, I 876

Kaaks, R 582

Kääriäinen, H 260, 732

Kaiser, G 1209

Kakizoe, T 678

Kakkar, AJ 876

Kalbakis, K 763

Kameoka, S 1168

Kamimura, M 1341

Kan, C-Y 946

Kanazawa, T 1317

Kanfer, EJ 620

Karim, R 364

Kasahara, T 544

Kaspers, GJL 1388

Katabuchi, H 116

Kataoka, K 678

Katis, C 1106

Kato, T 1168

Katsumata, N 999

Kazama, Y 1317

Kelland, LR 1011

Kelley, EA 1364

Kelly, J 1152

Kelsey, KT 949

Kennedy, JE 890

Key, T 1305

Key, TJ 582

Khal, J 774

Khaw, K-T 582

Khoo, M-L 279

Kida, A 884

Kiely, M 622

Kim, DH 1117

Kim, JC 1117

Kim, JG 1117

Kim, Y-I 639

Kimura, T 1168

Kinlen, L 963

Kirkpatrick, JP 1350

Kitade, Y 441

Kitagawa, R 999

Kiyono, T 116

Klæboe, L 842

Klagsbrun, M 233

Klapper, W 939

Kleinerman, R 260, 732

Klenk, J 1062

Kliewer, EV 159

Kneba, M 939

Knight, A 1215

Knoop, A 627

Ko, AH 195

Kobayakawa, M 1329

Kobayashi, K 1341

Kobayashi, M 1168

Kodera, Y 799, 986

Kohno, I 999

Koike, M 799

Koizumi, F 678

Kolberg, H-C 137

Kölby, L 1144

Kondo, M 557

Kono, N 884

Kooy, H 849

Koriakine, O 1209

Kornek, GV 744

Koskela, P 834

Kostrzewa-Nowak, D 89

Kotaniemi-Talonen, L 862

Kraunz, KS 949

Krogh, V 582

Kruger, S 137

Kruhoffer, M 464

Kudo, K 1341

Kuik, DJ 1388

Kuljaca, S 310

Kullmann, F 190

Kuramoto, H 999

Kuriu, Y 131

Kurozawa, Y 607

Kushida, M 441

Kvalheim, G 749

Kwan, ML 379

Kyte, JA 749

Lacombe, S 1098

Ladenstein, R 412

Lagarde, N 107

Lagergren, J 859

Lagiou, P 582

Lahmann, PH 582

Lai, MK 1057

Lamanna, G 633

Lang, F 744

Langridge, C 1092

Lankester, KJ 979

Laplanche, A 909

Lappin, TRJ 498

Larsen, S 273

Larsson, R 483

Larsson, SC 1310

Laud, PJ 208

Laugé, A 730

Lavarello, A 7

Lawes, DA 472

Lawrance, J 757

Lawson, JS 946

Layer, D 1202

Le Jeune, I 905

Leach, M 876

Leach, MO 979

Lee, HS 1038

Lee, KB 1117

Lee, M 310

Leek, RD 1168

LeGoffic, A 450

Lehtinen, M 834

Lencioni, M 35

Leonard, C 1364

Leonard, R 1215

Leong, S-S 23, 279

Lessard, L 1019

Leung, GM 1077

Levin, RD 1202

Levin, WP 849

Levitt, NC 60

Lewis, NR 905

L’Heureux, DZ 662

Li, CI 1046

Li, Y-Y 458

Lichtinghagen, R 565

Liff, JM 364

Lim, D 279

Lim, S-T 23

Lim, W-T 23

Lin, C-C 602

Lin, H-J 1372

Lin, J 1372

Lin, RS 1057

Lindmark, F 493

Lis, CG 1202

Liu, L 124

Liu, M 949

Liu, T 310

Liu, W 233

Lizón, J 1230

Llambi, F 1

Loddo, M 1295

Loeffler, JS 849

Long, J 620

Lönn, S 842

Lopez, JG 242

Lopez, M 896

López-Gómez, L 1230

Lord, SJ 364

Lordick, F 190

Lorenzen, S 190

Lorigan, PC 620

Lorusso, V 29

Lövborg, H 483

Lozac’h, P 107

Lu, P 279

Lu, Q 662

Lucchi, M 453

Ludovini, V 1334

Luke, BT 953

Luostarinen, T 834

Ma, MK 46

Machin, D 279

Mäcke, H 1144

MacManus, M 652

Madhusudan, S 60

Maeda, T 116

Magistrelli, P 1084

Magnusson, CMK 817

Magrini, E 1334

Mahoney, M 699

Maisey, NR 740

Malacrino, C 1084

Man, S 1068

Manabe, T 355

Mancini, E 376

Mann, CH 248

Mansoor, W 757, 1085

Manzione, L 611, 896

Marano, O 611

Marchbanks, PA 364

Marcon, N 639

Marcussen, N 1182

Margison, GP 529, 1152

Marini, L 29, 406

Markakis, E 152

Marosis, C 1106

Marshall, E 647, 1324

Marshall, GM 310

Martin, NMB 1011

Martos, C 159

Marubashi, S 557

Maruyama, R 544

Marzano, N 611

Masi, A 781

Mason, M 178

Massey, A 740

Massidda, B 185

Massion, PP 1157

Mataki, Y 688

Matakidou, A 825

Mathot, RAA 173

Matsumoto, T 331

Matsumura, Y 678

Matsuo, K 15

Mattioli, R 185

Matulonis, UA 54

Maughan, T 993

Maur, M 29

Mavroudis, D 763

McArdle, CS 977

McBride, ML 159

McCarty, MF 233

McClory, B 1112

McElhinney, RS 1152

McGhee, SM 1077

McKendrick, J 652

McKinney, PA 842

McLeod, HL 46

McMillan, DC 977

McMurry, TBH 1152

McNeilly, A 647

McPherson, K 817

McQuaid, D 699

McShane, LM 387

Mead, GM 178

Meadows, HM 993

Mehlen, P 1

Meijerink, JPP 1388

Mel, JR 1230

Meldgaard, P 757

Mellado, B 1285

Mellemkjaer, L 159, 838

Mellor, HR 302

Mermelshtein, A 338

Mes-Masson, A-M 1019

Metayer, C 379

Metges, J-P 107

Meusers, P 939

Mey, V 35

Meyers, DA 493

Michael, M 652

Michaelson, JS 1244

Michalopoulou, P 1106

Michaud, DS 804

Middleton, M 757

Middleton, MR 890

Migliaccio, I 464

Mikula, M 144

Mileshkin, L 652

Millar, J 1112

Miller, ID 1301

Milner, AD 652

Minkin, S 639

Minna, JD 1029

Mitchell, F 868

Miura, Y 441

Miyamoto, A 557

Moan, J 571

Mochizuki, Y 986

Modjtahedi, H 1316

Mognetti, B 1250

Møller, S 627

Monden, M 557

Montanari, G 376

Moore, MJ 1236

Morgan, GJ 269, 811

Morison, L 1068

Morris, JS 804

Morrison, A 1112

Mortimer, JE 46

Mosca, A 633

Mosca, F 35

Moscetti, L 185

Motoyama, T 1395

Moynihan, C 200

Moysich, K 699

Mu, LJ 749

Muir, K 842

Mullamitha, S 647

Mumoli, N 266

Murphy, D 248

Murphy, MFG 597

Murray, GI 1301

Musholt, PB 565

Musholt, TJ 565

Mussi, A 453

Myllymäki, L 435

Naccarato, AG 406

Nagano, H 557

Nagawa, H 1317

Naito, S 678

Naka, G 1341

Nakamori, S 557

Nakamura, M 557

Nakanishi, H 986

Nakanishi, Y 1382

Nakao, A 799

Näslund, I 1310

Nasti, G 611

Natsugoe, S 688

Neale, RE 597

Negishi, T 678

Nellemann, H 757

Nelson, HH 949

Nerusu, KC 1364

Newlands, ES 620

Newton, R 1305

Nguyen, T 310

Nicholls, E 200

Nicolò, G 7

Nieminen, P 862

Nikodemska, A 144

Nikolakopoulos, J 763

Nilsson, BR 124

Nilsson, O 1144

Nilsson, UW 1005

Nishigami, T 331

Nishikawa, M 331

Nishikawa, T 1168

Nishimura, R 999

Nishimura, T 131

Nishiyama, N 678

Nishiyama, T 544

Nishizuka, S 1395

Noda, K 999

Nordestgaard, BG 167

Norman, A 200

Norman, AR 510, 740

Norman, SA 364

Norris, MD 310

Nose, T 607

Nouyrigat, E 909

Novello, S 29

Nowak, NJ 699

Nozawa, S 999

Nutley, BP 1011

Nuyten, DSA 924

Nuzzo, G 1084

Nygren, P 483

Oates, J 510, 740

Oberaigner, W 1062

O’Donnell, A 876

O’Dwyer, PJ 1356

Ogimoto, I 607

Ohashi, M 441

Ohmori, M 1202

Ohnami, S 441

Ohradanova, A 1267

Ohtake, H 116

Okamoto, K 131

Okamura, H 116

Okines, A 418

Olsen, A 582

Olsen, JH 260, 732, 838, 1038

Onganer, PU 1197

O’Quigley, J 529

Ord, JJ 346

Orlandi, P 319

Orlandini, C 406

Orlowska-Volk, M 694

Ørntoft, TF 464, 1182

Osada, H 1157

Oshitani, N 331

Ostrowski, J 144

Ota, H 557

Otsuji, E 131

Otter, R 520

Ottesen, LH 915

Overvad, K 582

Padhani, A 876

Padhani, AR 979

Paglierani, M 781

Paine, MJI 89

Palazzo, S 896

Pallante, P 464

Palmeri, S 896

Palombini, L 464

Paluchowska, B 1209

Pang, X-W 458

Paoletti, G 896

Pappagallo, G 611

Park, IK 1117

Park, JS 1117

Parker, C 200, 478

Parkin, DE 1301

Parwaresch, R 939

Passagne, I 216

Pastorek, J 1267

Pastorekova, S 1267

Patriarca, S 376

Pearson, T 472

Pedersen, L 838

Pedersen, MW 915

Pedersen, N 915

Pedicini, T 611

Pedotti, P 1053

Pein, F 529

Pelegrín, A 1230

Pemberton, MN 208

Penson, RT 54

Perdelli, L 7

Pericleous, LM 1257

Permert, J 1310

Perrin, P 242

Perrone, F 896

Persico, G 896

Peschel, C 190

Peschos, D 1382

Peters, GJ 1388

Peters, TJ 399

Peterse, JL 924

Peterson, C 1202

Petitti, DB 392

Peto, J 817

Petrich, T 565

Pezzella, F 1168

Pezzella, G 611

Philips, H 1112

Phillips, RR 890

Phillips, S 876

Piaton, E 242

Picciocchi, A 1084

Picus, J 46

Pieters, R 1388

Pike, MC 817

Piketty, A-C 909

Pilutti, S 376

Pinot, D 450

Pisano, A 611

Pisconti, S 611

Pistillucci, G 896

Pizza, C 611

Pluim, D 1222

Poikonen, P 515

Pollo, B 781

Polvani, S 781

Polyzos, A 763

Pompe-Kirn, V 159

Porojnicu, AC 571

Porpiglia, F 633

Potter, E 565

Poulsen, HS 915

Prati, MC 453

Prengel, C 1175

Press, MF 364

Prevo, R 1168

Prevost, AT 1295

Price, A 1112

Price, TJ 510

Prise, VE 98

Propper, DJ 60

Protheroe, AS 890

Pryshchepava, A 699

Przytul-a, E 144

Pu, YS 1057

Pujol, J-L 1098

Pukkala, E 159

Pyle, L 868

Qiao, G-B 137

Qiao, H 458

Quattrin, S 611

Quiton, J 1202

Raabe, N 749

Rabbani, ZN 1350

Radstone, CR 418

Raguz, S 973

Raimondi, S 1053

Raitanen, J 842

Ramani, V 670

Randall, KJ 208

Rao, S 510

Rapp, K 1062

Rapti, A 763

Raquin, M-A 529

Ratner, L 46

Ratschiller, D 719

Ravazoula, P 1382

Raymond, DD 662

Raynaud, F 868, 876

Raynaud, FI 1011

Recchia, F 185

Rechnitzer, C 1038

Reddy, M 1364

Ree, AH 571

Rees, GS 1038

Reeves, G 590

Restano-Cassulini, R 781

Ribeiro, T 1305

Riboli, E 582

Ricci, S 35

Richards, J 1257

Rickardson, L 483

Rischin, D 652

Risk, JM 960

Robaszkiewicz, M 107

Roberts, H 1168

Roberts, ISD 346

Roberts, S 248

Robsahm, TE 260, 571, 732

Roche, M 54

Roddam, AW 817

Rollason, T 248

Roman, E 811

Roncella, M 406

Ronco, G 376

Rondini, M 406

Rookes, S 248

Rookus, MA 287

Rooney, PH 1301

Ross, JS 1285

Rossi, E 1334

Rosso, R 7, 733

Rosti, G 412

Round, A 399

Rovira, A 1285

Rubie, H 529

Ruddle, R 868

Ruffion, A 242

Ruiz, M 1230

Russell, SEH 499

Russell, ST 425, 774

Rustin, GJS 178, 979

Rutgers, EJT 287

Rydåker, M 483

Saad, F 1019

Sabherwal, Y 662

Sainsbury, R 1295

Sakakibara, T 799

Sakata, R 607

Sakon, M 557

Salminen, T 842

Salud, A 1230

Samonis, G 763

Sanders, PM 425

Sankila, R 260, 732

Santibáñez-Koref, MF 719

Santini, D 1084

Santoro, A 406

Santoro, M 453, 464

Saracchini, S 406

Sargeant, A-M 1236

Sauerbrei, W 387

Sauleda, S 1285

Saunders, C 1319

Scagliotti, GV 29

Scarpa, RM 633

Scarpa, S 1084

Scélo, G 159

Scetbon, F 1175

Schaapveld, M 520

Scheithauer, W 744

Schellens, JHM 1222

Scherf, CF 1068

Schmidt, H 273

Schmidt, MK 287

Schmitt, A 1144

Schneeweiss, B 744

Schoemaker, MJ 842

Schrader, C 939

Schrøder, H 1038

Schroeder, J 1062

Schuell, B 744

Schulz, M 582

Scopa, CD 1382

Scorilas, A 152

Scullin, P 1112

Scurr, M 876

Sebag-Montefiore, D 993

Seckl, MJ 620, 1197

Sedlakova, O 1267

See, A 279

Segnan, N 376

Seiden, MV 54

Selby, P 622

Selvakumaran, M 1356

Selvin, S 379

Semba, H 770

SenGupta, S 472

Senn, H-J 793

Seow, A 159

Seroneit, T 190

Seto, T 770

Sgroi, D 1244

Shames, DS 1029

Shan, S 1350

Shannon, AM 224

Sharma, P 1202

Sharp, D 399

Shaw, RJ 960

Shechter-Maor, G 338

Sheng, X 1038

Shepherd, L 1236

Shetty, A 1295

Shi, Y 1277

Shibata, A 607

Shida, S 1356

Shieh, T-Y 602

Shigematsu, H 1029

Shishodia, S 70

Shivapurkar, N 1029

Siebmann, J-U 939

Siegel-Lakhai, WS 1222

Silverstein, MJ 1244

Simes, J 1236

Simes, RJ 293

Simmons, L 868

Simon, MS 364

Sjöström, J 515

Skibola, CF 811

Skibola, DR 811

Skov, L 538

Skriver, MV 838

Skubis-Zegadl-o, J 144

Slavin, S 412

Smart, V 1038

Smith, A 622

Smith, B 1244

Smith, GCM 1011

Smith, MT 811

Smith, NF 1011

Smith, S 310

Smith, SL 719

Snyder, SA 1350

Sobrero, A 733

Sohda, Y 999

Sohn, JH 1117

Sohn, SK 1117

Solanki, B 1222

Solomon, B 652

Somcio, R 233

Song, H 1372

Sonobe, M 355

Soo, K-C 279

Sørensen, HT 838

Spadaro, M 1250

Sparidans, RW 1222

Sparreboom, A 173

Spirtas, R 364

Stam, RW 1388

Stathopoulos, GP 1106

Stathopoulos, J 1106

Stayerman, C 552

Steele, JC 248

Steers, G 1168

Stenning, SP 178

Stenram, U 435

Stern, PL 670

Steven, N 1324

Stickeler, E 694

Stirling, D 613

Stirling, JJ 979

Stockler, MR 1319

Stoeber, K 1295

Stoehr, S 1062

Stoeltzing, O 233

Stollfuss, J 190

Stoppa-Lyonnet, D 730

Straume, O 933

Streeter, EH 346

Strom, BL 364

Stuerzbecher, H-W 137

Sugita, M 884

Sun, J 493

Sunaga, N 1029

Sung, F-C 1057

Susumu, N 999

Suzuki, H 607

Suzuki, M 1029

Svastova, E 1267

Swerdlow, AJ 842

Syrigos, K 763

Tada, T 1317

Taddei, GL 781

Tai, B-C 279

Taioli, E 1053

Tajima, K 15

Takahashi, K 544

Takahashi, T 1029, 1157

Takeda, Y 1341

Talieri, M 152

Talpaz, M 70

Tamakoshi, A 607

Tamanoi, M 770

Tampellini, M 633

Tamura, G 1395

Tan, BR 46

Tan, E-H 23, 279

Tan, T 279

Tanabe, KK 1244

Tanaka, F 355, 770

Tanaka, J 1317

Tanaka, T 1317

Tarabuzzi, R 633

Tarasiuk, J 89

Tari, M 81

Tashiro, H 116

Tatematsu, M 986

Taube, SE 387

Tawn, EJ 1038

Tay, M-H 23

Taylor, NJ 979

te Poele, R 60

Tebbutt, N 510

Tedeschi, R 834

Tempero, MA 195

Tenkanen, L 834

ter Haar, GR 890

Terashima, M 1395

Terrone, C 633

Testa, J 1356

Testore, F 7

Thach, TQ 1077

Thatcher, N 757

Théodore, C 909

Thistlethwaite, FC 1085

Thoedtmann, J 190

Thomas, E 960

Thomsen, J 842

Thomson, CS 418

Thykjaer, T 1182

Tiemann, M 939

Tisdale, MJ 425, 774

Tjønneland, A 582

Toh, C-K 23

Tokudome, S 15

Tokudome, Y 15

Tollenaar, RAEM 287

Toma, A 131

Tomita, Y 544

Ton, N 647

Tonini, G 1084

Tonita, JM 159

Toomey, D 224

Torre, J-P 450

Tortoriello, A 611

Toschi, L 1334

Touzé, A 1305

Toyama, T 15

Tozer, A 613

Tozer, GM 98

Tracey, E 159

Tralongo, P 185

Tranberg, K-G 435

Trangas, T 152

Tretli, S 260, 732, 807

Trichopoulos, D 582

Trichopoulou, A 582

Trigo, JM 876

Trione, E 1250

Troncone, G 464

Truan, D 1285

Tsavdaridis, D 1106

Tselepatiotis, E 763

Tsuduki, E 1341

Tsukuda, M 884

Tu, D 1236

Tucci, M 633

Tucker, M 260, 732

Tufto, I 81

Tumolo, S 406

Turley, H 1128

Tuszynski, GP 662

Tybjærg-Hansen, A 167

Tynes, T 842

Uchino, H 678

Uchitomi, Y 1329

Ueki, K 999

Ueki, M 999

Uenosono, Y 688

Ulmer, H 1062

Umeshita, K 557

Ural, AU 267

Uribe, DJ 1046

Ursin, G 364

Utzmann, O 1175

Væth, M 538

van Asperen, CJ 287

van Dam, P 1128

Van den Eynden, GG 1128

Van der Auwera, I 1128

Van Laere, SJ 1128

Van Marck, EA 1128

van Oosterom, AT 1209

van Sprundel, TC 287

van ’t Veer, LJ 924

van Wering, ER 1388

Van, Q 70

Vana, F 633

van‘t Veer, LJ 287

Varani, J 1364

Varella-Garcia, M 1334

Varma, G 699

Varma, R 699

Varner, JA 855

Vasaturo, T 1084

Vassal, G 529

Vehling-Kaiser, U 190

Velikova, G 622

Venook, AP 195

Venturini, M 7

Vermeulen, PB 1128

Verweij, J 173

Vessey, M 817

Viacava, P 406

Vian, L 216

Viglietto, G 464

Villman, K 515

Vincenzi, B 1084

Virtamo, J 834

Volant, A 107

Volante, R 376

von der Maase, H 273, 757

von Moos, R 793

von Wasielewski, R 565

Vujaskovic, Z 1350

Wada, H 355, 557

Waehre, H 749

Wakai, K 15

Wakasa, K 557

Walfisch, S 338

Wall, L 1112

Wall, SR 1068

Wallam, M 1202

Walraven, G 1068

Walt, H 1137

Wang, H-C 458

Wang, M 70

Wanke, E 781

Wartelle, M 529

Watanabe, T 1317

Watermann, DO 694

Watson, AJ 529, 1152

Watson, K 1202

Weber, D 70

Wee, J 279

Wehmeier, M 565

Weigelt, B 924

Weiland, SK 1062

Weiss, LK 364

Wenzel, BE 144

Wessels, LFA 924

West, B 1068

Wey, JS 233

Whitaker, NJ 946

Whitehouse, CA 1038

Whiteman, DC 597

Wiemels, JL 379

Wiencke, JK 949

Wiggers, T 1122

Wiklund, F 493

Wilcken, N 293

Willemse, PHB 520

Willett, EV 811

Williams, GH 1295

Wilson, E 41

Wilson, I 98

Winterbottom, A 622

Winther, JF 1038

Wirfält, E 582

Wirth, A 652

Wolf, CR 89

Wolk, A 1310

Wong, E-H 23

Woo, PPS 1077

Wood, PA 1202

Workman, P 876, 1011

Wright, LP 46

Wright, P 622

Wu, F 890

Wu, Y-L 137

Wyke, SM 774

Xie, D 137

Xu, J 493

Yagüe, E 973

Yamagishi, H 131

Yamaguchi, S 999

Yamamoto, H 770

Yamamoto, T 557

Yamamura, Y 986

Yamana, K 544

Yamawaki, S 1329

Yanagida, S 688

Yang, X-A 458

Yang, X-N 137

Yang, Y-H 602

Yaniv, I 412

Yannopoulos, A 152

Yao, K 1356

Yin, Y-H 458

Yoshida, K 441

Yoshida, T 441

Yoshihara, T 884

Yoshimura, T 607

Yuen, J 260, 732, 1236

Zachariae, C 538

Zachariae, R 538

Zanetti, R 376

Zatovicova, M 1267

Zeldis, JB 613

Zhang, H-G 458

Zhang, S 1222

Zhang, Y 458

Zheng, SL 493

Zhong, W-Z 137

Zidan, J 552

Zou, M 1277

Zumschlinge, R 190

zur Hausen, A 694

Zwaan, CM 1388

Zwahlen, D 709

